# Increased population sampling confirms low genetic divergence among Pteropus (Chiroptera: Pteropodidae) fruit bats of Madagascar and other western Indian Ocean islands

**DOI:** 10.1371/currents.RRN1226

**Published:** 2011-03-21

**Authors:** Lauren M. Chan, Steven M. Goodman, Michael D. Nowak, David W. Weisrock, Anne D. Yoder

**Affiliations:** ^*^Department of Biology, Box 90338, Duke University, Durham, NC 27708; ^†^Field Museum of Natural History, 1400 South Lake Shore Drive, Chicago, IL 60605, USA, and Vahatra, BP 3972, Antananarivo 101, Madagascar; ^‡^Centro de Ciências do Mar do Algarve, Universidade do Algarve, Campus de Gambelas 8005-139, Faro, Portugal and ^§^Department of Biology, University of Kentucky, 101 TH Morgan Building, Lexington KY 40506

## Abstract

Fruit bats of the genus Pteropus occur throughout the Austral-Asian region west to islands off the eastern coast of Africa. Recent phylogenetic analyses of Pteropus from the western Indian Ocean found low sequence divergence and poor phylogenetic resolution among several morphologically defined species. We reexamine the phylogenetic relationships of these taxa by using multiple individuals per species. In addition, we estimate population genetic structure in two well-sampled taxa occurring on Madagascar and the Comoro Islands (P. rufus and P. seychellensis comorensis). Despite finding a similar pattern of low sequence divergence among species, increased sampling provides insight into the phylogeographic history of western Indian Ocean Pteropus, uncovering high levels of gene flow within species.

## Introduction

    Flying foxes, belonging to the family Pteropodidae (Genus *Pteropus*), are species rich with 65 recognized species primarily distributed from Australia, westwards though tropical and subtropical Asia, to islands in the western Indian Ocean and offshore eastern Africa [1]. All of these species have been described based on morphological characters, including size, pelage coloration, and cranio-dental characters. Resolving the phylogenetic relationships among species, typifying patterns of gene flow within members of the genus, and identifying instances of hybridization among species is important both for taxonomy and phylogeography; these aspects in turn provide insights for conservation. Fruit bats, including * Pteropus*, have been identified as reservoirs for pathogens known to cause zoonotic diseases in humans [2] [3] [4]; *Pteropus* bats come into close contact with human populations through their utilization as bush-meat and the consumption of fruits from the same trees [5]. Data on connectivity within and among populations are critical for understanding aspects of disease transmission.

    Certain volant mammals, including members of the genus *Pteropus*, are capable of long distance dispersal [6].  Several * Pteropus* species are known to travel up to 50 km per night between day roosts and feeding sites and they are capable of traversing considerable expanses across open water (e.g., [7] [8]). For example, based on satellite telemetry, *P. vampyrus* is notably mobile traveling hundreds of kilometers between day-roost sites during the course of a year, and crossing international borders [9]. Past studies have found varying levels of intraspecific genetic differentiation among island populations of pteropodids based on mitochondrial sequences and allozyme data. For some members of the genus *Pteropus, * there is considerable gene flow within species across broad geographical areas, but very little differentiation between subspecies or sister species [10] [11] [12]. In contrast, other genera of Pteropodidae from southwest Pacific Ocean islands tend to show greater levels of intraspecific genetic structure [10] [13].

    There are eight recognized species of *Pteropus * on offshore islands along the coast of eastern Africa and in the western Indian Ocean, including Madagascar and the Mascarene, Seychelles, and Comoro archipelagos (Figure 1)**.**

**Figure:**
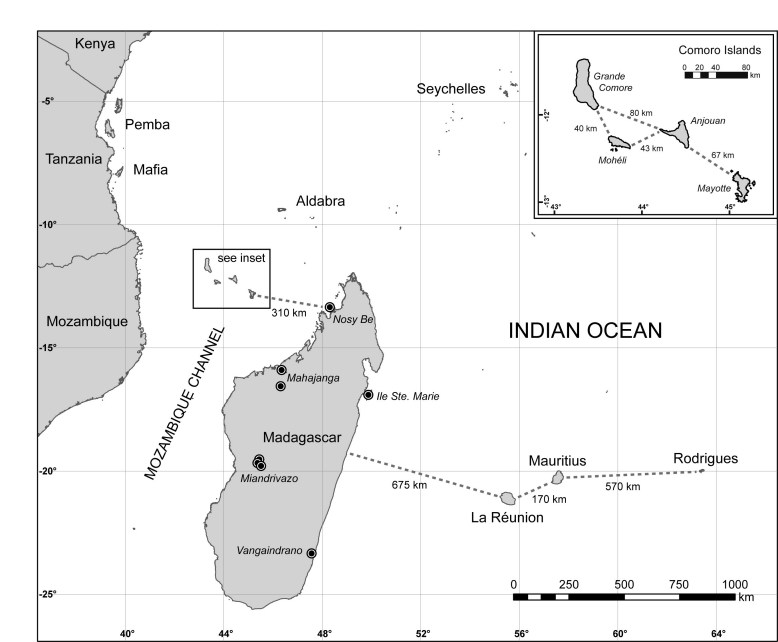
**Figure 1.**Map of the western Indian Ocean islands. Collection localities of *P. rufus* on Madagascar are indicated with circles and the five names associated with the grouped samples are indicated in italics.

Many*Pteropus* taxa are restricted to a single island or portion of an archipelago [14] [15] [16] and are diagnosed based on morphological characters. For example, *P. seychellensis comorensis* from the Comoros are considered to be closely related to *P. s. seychellensis* on the distant Seychelles based on size and pelage [1], rather than to the more geographically proximate * P. rufus *in Madagascar (Figure 1).

    Recently, O’Brien et al. [15] investigated the molecular systematics and associated phylogeography of *Pteropus* from the Indian Ocean. They found strong support for clades within *Pteropus*, including the resolution of a clade containing *P. rufus * from Madagascar, the subspecies *P. s. seychellensis* and *P. s. comorensis*, *P. aldabrensis* from Aldabra Atoll (extreme western Seychelles)and *P. niger* from Mauritius (Figure 1). However, within this clade, there was low support for the monophyly of any of these species and for the relationships among these taxa (Figure 2).

**Figure:**
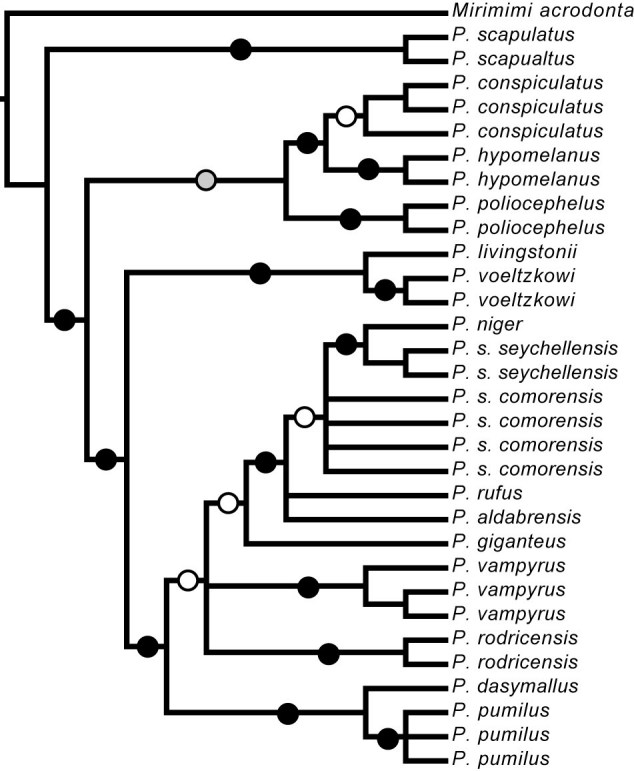
**Figure 2. **Cladogram of phylogenetic relationships of different *Pteropus *species recovered by O’Brien et al. [15] for 12S+Cyt-*b*. Black circles indicate nodes with strong support in MP bootstrap, ML bootstrap, and Bayesian phylogenetic analyses (≥ 75 bootstrap value, ≥ 0.95 posterior probability). Grey and white circles denote nodes with strong support in two of the three analyses and one of three analyses, respectively.

This suggests that there may be recent or current gene flow among species and calls into question the current taxonomy of regional members of this genus. While sampling within this clade was limited, *P. s. seychellensis *and *P. s. comorensis * were not recovered as sister taxa. Instead, *P. s. seychellensis* was strongly supported as sister to *P. niger* (constrained to be monophyletic in their analyses) and this clade formed an unresolved polytomy with multiple samples of *P. s. comorensis* and single representatives of both *P. rufus*, and *P. aldabrensis.*

    Here, we re-evaluate the phylogeographic history and estimate the population genetic structure of western Indian Ocean *Pteropus*. Our aims are twofold. First, we investigate whether increased sampling helps to clarify species limits and elucidate phylogenetic relationships among * P. rufus*,* P. s. comorenesis,* *P. s. seychellensis*,* P. niger*,and *P. aldabrensis. * Second, we use population sampling to examine patterns of genetic connectivity within *P. rufus* on Madagascar and *P. s. comorensis* on the Comoros.

## Materials and Methods

    Ear clips were collected in the field from released individuals in the context of a research program of the Institut Pasteur de Madagascar and preserved in EDTA. Additional tissue samples associated with specimens housed in the Field Museum of Natural History and from captive animals at the Lubee Bat Conservancy were also included (Table 1).

**Table 1.** Newly sequenced samples included in this study. Abbreviations as follows. CS: Institut Pasteur; Chauve-souris; SMG: Steven M. Goodman; ISIS: Lubee Foundation; AJ, AJL, R, H, GC, MH, MT, and V: Lubee Bat Conservancy band identification numbers.

**Table:** 

**Species**	**Sample ID**	**Locality**
*Pteropus giganteus*	ISIS930045	Lubee Bat Conservancy, Captive Born
*Pteropus hypomelanus*	H10	Lubee Bat Conservancy
*Pteropus livingstonii*	AJL07-11, AJL14-17, AJL19	Comoro Islands, Anjouan
*Pteropus poliocephalus*	ISIS930315, ISIS930139-930141, ISIS930349, ISIS930372, ISIS930375	Lubee Bat Conservancy, Captive Born
*Pteropus rodricensis*	R20-21	Lubee Bat Conservancy
*Pteropus rufus*	CS117-126	Madagascar: Province de Toliara, Boromena, Miandrivazo
	CS132-137, CS141-142	Madagascar: Province de Toliara, Ankotrofotsy, Miandrivazo
	CS230, CS285, CS516, CS518, CS521-522	Madagascar: Province de Fianarantsoa, Vangaindrano
	SMG14638-14647	Madagascar: Province de Mahajanga, near Maroadabo
	SMG14737-14746	Madagascar: Province de Mahajanga, near Andakalaka
	SMG15042-15044	Madagascar: Province d'Antsiranana, Nosy Be
	SMG15295-15304	Madagascar: Province de Toamasina, Isle Sainte Marie
*Pteropus seychellensis comorensis*	AJ05, AJ08, AJ23-27, AJ28A, AJ42, AJ44	Comoro Islands, Anjouan
	GC06a,GC07a, GC27a-29a, GC48a, GC50a, GC05b, GC08b	Comoro Islands, Grande Comore
	MH04a, MH06, MH07a-08a, MH27-28, MH35, MH37, MH41-42	Comoro Islands, Mohéli
	MT02a-05a, MT10-14, MT23	Comoro Islands, Mayotte
*Pteropus vampyrus*	V7	Lubee Bat Conservancy

    Genomic DNA was extracted from a small tissue sample using either the Nucleospin DNA Extraction Kit or the Qiagen DNeasy Tissue Kit. The mitochondrial gene, cytochrome-b (cyt-*b*), was amplified using the primers L14724 and H15915 [17]. PCR reactions were conducted in a total volume of 25 µL including 1 x Buffer (100 mM Tris-HCL, pH 8.3, 500 mM KCl), 2 mM MgCl_2_, 1 mM dNTP, 0.25 µM of each primer, 0.5 U *Taq* polymerase, and 1 µL template DNA. PCR cycles consisted of an initial denaturation of 94 °C for 2 min, 35 cycles of 94 °C denaturation for 1 min, 48 °C annealing for 1 min, and 72 °C extension for 1.5 min, and a final extension of 10 min at 72 °C. Five µL of amplified product were incubated with 0.4 µL ExoSapIT (USB) and 1.6 µL water at 37 °C for 15 minutes followed by 80 °C for 15 minutes. Cleaned PCR products were sequenced using the primers used for amplification and an internal primer when necessary (PterCytbInt1 5’ GGRGCAACAGTCATYACYAA 3’) in a total volume of 5 µL (1 x buffer, 1 µM primer, 0.2 µL BigDye v3, and 1.0 µL cleaned PCR product). Sequencing reactions were run on an ABI 3730xl DNA Analyzer capillary machine and sequences were checked by eye and assembled into contigs in Sequencher 4.8 (GeneCodes, Ann Arbor, Michigan). 

    We supplemented these data with sequences from GenBank (see Table S1) and used MacClade [18] to align sequences by hand and check for stop codons. All sequences have been deposited in GenBank (JF327207 – JF327326). We reconstructed the phylogenetic relationships among DNA sequences under maximum parsimony (MP), maximum likelihood (ML), and Bayesian inference using other Pteropodidae (*Ptenochirus* *jagori* and *Cynopterus brachyotis*) as outgroups to root phylogenies based on previous phylogenetic work [19] [20] [21] [22]. Maximum parsimony bootstrap analyses were conducted in PAUP 4b10; we used 1,000 bootstrap replicates of the fast-search option.

    We determined the model of sequence evolution that best fit the entire data set and each partition of the data (first, second, and third codon positions) using MrModeltest 2.3 [23] [24]. One hundred ML bootstrap replicates were conducted in Garli 1.0 under a GTR+I.+G model of sequence evolution. Partitioned Bayesian phylogenetic analyses were performed in MrBayes 3.1.2 [25] [26]. These consisted of three independent runs each with four incrementally heated chains sampled every 1,000 generations for 10 million generations. We verified adequate mixing within runs and convergence among runs in Tracer and discarded the first 1,001 samples from each run before summarizing trees.

    To examine phylogeographic structure within species, we constructed a haplotype network within a parsimony framework in TCS 1.21 [27]. We included all descendants of the most recent common ancestor for *P. rufus *and *P. s. comorensis* that were well-supported in the Bayesian phylogenetic inference. We used a 95% connection limit and treated gaps as a fifth state.

    We assessed whether genetic diversity was best explained by within population variation or among population variation using an AMOVA executed in Arlequin 3.5 [28]. Samples of *P*.* s*.* comorensis* from the Comoros were grouped by island (Grande Comore, Mohéli, Anjouan, and Mayotte) and *P. rufus *from Madagascar were separated into five groups based on the proximity of collection localities (Figure 1).

## Results

    The final cyt-*b* sequence alignment consisted of 1,123 base pairs for 167 individuals. Across all samples, including outgroups, we found 648 invariable and 375 parsimony informative sites. Among *Pteropus* sequences, 755 sites were invariable and 294 were parsimony informative. We found 33 unique haplotypes among *P. rufus * and 12 unique haplotypes among *P. s. comorensis*.

    Phylogenetic analyses of cyt-*b* sequence data using three optimality criteria were largely congruent at deeper nodes, but support values for relationships within *Pteropus* were not always consistent across analyses. * Pteropus* was resolved as a monophyletic group with low support (Figure 3). 

**Figure:**
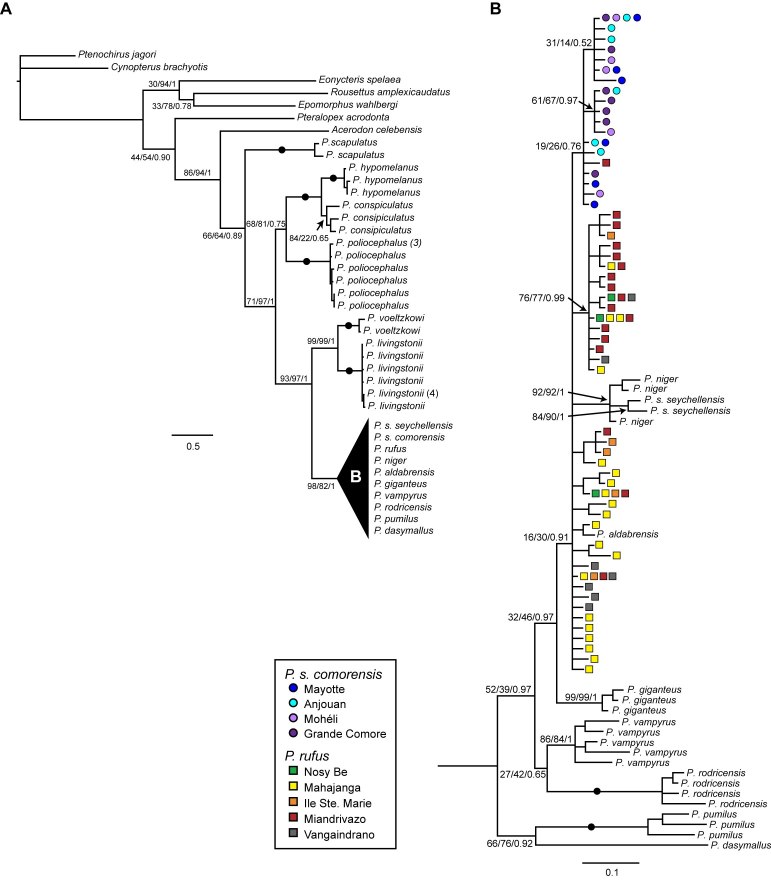
**Figure 3.** Majority-rules consensus tree from Bayesian analyses with MP bootstrap, ML bootstrap, and Bayesian support values at each node. Circles at the node indicate maximum support in all three analyses.

We found moderate to strong support for the monophyly for a group containing all *Pteropus* except *P. scapulatus* (Figure 3A). Among western Indian Ocean taxa, our data support the sister species relationship between *P. livingstonii* from Anjouan (Comoros), and *P. voeltzkowi*, from the Pemba Island (Tanzania) (Figure 1). There was little resolution within a clade containing four species of western Indian Ocean *Pteropus* (*P. aldabrensis, P. niger, P. rufus, *and* P. seychellensis*) (Figure 3B). We did not find any support for the monophyly of *P. rufus * (MP=0; ML=0; BP=0)and very weak support for the monophyly of * P. s. comorensis (*MP=23; ML=0; BP=0.16). Moreover, there was no support for a sister pair relationship between the two races of *P. seychellensis *(MP=0; ML=0; BP=0), but we did find strong support for a clade comprising *P. s. seychellensis* and *P. niger*. 

    Mitochondrial haplotypes from each species clustered together in the haplotype network, but *P. s. comorensis* and *P. rufus* alleles differed by as few as two base-pairs (Figure 4). Based on this haplotype network, * P. s. seychellensis* was more closely related to *P. niger* and together, were more closely related to *P*. *rufus* than to *P*.* s*.* comorensis*.

**Figure:**
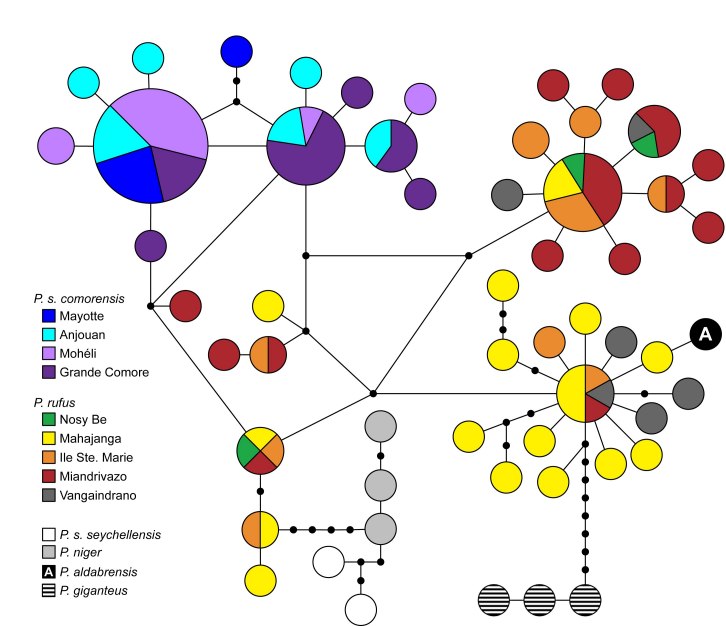
**Figure 4.** Haplotype network of individuals from multiple localities for *Pteropus rufus* and *P. s. comorensis,* as well as individuals from *P. s. seychellensis*,* P. aldabrensis*,* P. giganteus*,* *and* P. niger*. The size of the circle indicates the number of individuals with that haplotype with the frequency of each haplotype across multiple localities indicated with pie charts.

    We found evidence for population genetic structure in *P*.* s*.* comorensis* and *P. rufus*, although values of ϕ_ST_ and F_ST_ were low. For *P*.* s*.* comorensis*, overall ϕ_ST_ was 0.0676 and was significant at p = 0.0423; however, in pairwise population tests of differentiation, only one pair of islands had an F_ST_ significantly greater than zero (Table 2). In contrast overall ϕ_ST_ for *P. rufus * was 0.130 (p < 0.001). Three pairwise population F_ST_ values were significantly different from zero, but these did not show a clear trend with geographic distance (Table 3).

**Table 2.** F_ST_ for *P*.* seychellensis comorensis*. Estimates of F_ST_ significantly different from zero are indicated in bold.

**Table:** 

	Grande Comore	Mohéli	Anjouan
Mohéli	0.12936
Anjouan	-0.02970	0.04542
Mayotte	0.09878	**0.14665**	-0.00355

**Table 3.** F_ST_ for *Pteropus rufus.* Estimates of F_ST_ significantly different from zero are indicated in bold.

**Table:** 

	Mahajanga	Nosy Be	Ile Ste Marie	Miandrivazo
Nosy Be	0.09955
Ile St Marie	0.05057	-0.09239
Miandrivazo	**0.25611**	-0.14147	**0.08397**
Vangaindrano	-0.01234	0.03226	0.02564	**0.18684 **

## Discussion

    By sampling multiple individuals per species, we are able confirm the monophyly of several taxa where sampling was previously limited [15], including *P. hypomelanus*, *P. poliocephalus*, * P. livingstonii*, *P. rodricensis*, *P. vampyrus*, and * P. giganteus *(Figure 3). However, increased sampling did not help to resolve the interspecific relationships at deeper nodes in the phylogeny, and in this sense, have not improved upon those of O’Brien et al. [15]. In general, the phylogenetic relationships among *Pteropus* species were more strongly supported in Bayesian analyses than in MP or ML analyses. A monophyletic group containing four western Indian Ocean species (*P. rufus, P. niger, P. aldabrensis, *and *P. seychellensis*) was suggested by Bayesian inference, but did not receive strong support in either MP or ML analyses (Figure 3). Within this group, we found support for the monophyly of *P. s. seychellensis*, but not for any other species.

    Population sampling for *P. rufus* and *P. s. comorensis * confirms that interspecific sequence divergence is low and additionally suggests that *P. rufus* may be paraphyletic with respect to * P. s. comorensis* and *P. aldabrensis*. We did not find support for the monophyly of either *P. rufus * or *P. s. comorensis* and recovered weak support for a clade containing all *P. s. comorensis *haplotypes and one *P. rufus* haplotype from Miandrivazo (Figure 1, 3). It is possible that this phylogenetic pattern simply reflects a lack of resolution due to low mitochondrial sequence divergence or incomplete lineage sorting. However, if the * P. rufus* individual is truly most closely related to *P. s. comorensis*, migration from the Comoros to Madagascar with subsequent introgression may be an alternative explanation for this pattern.

    We included multiple samples of *P. niger* but in constrast to O’Brien et al. [15] we did not constrain them to be monophyletic. While we also found that *P. s. seychellensis* and *P. niger* are most closely related to one another and that *P. s. * seychellensis is not sister to *P. s. comorensis*, we were unable to determine whether the former two taxa are reciprocally monophyletic and the position of these two species relative to *P. rufus*,* P. s. comorensis*, and *P. aldabrensis* remains unclear. Our results place, *P. niger - P. s. seychellensis * as part of a larger polytomy that includes the latter three species (Figure 3), but these haplotypes are most closely related to *P. rufus* in parsimony networks (Figure 4).

    Low sequence divergence within this clade of western Indian Ocean *Pteropus * suggests that these species diverged very recently. The island of Madagascar is at least 80 ma, considerably older than the islands and archipelagos of Mauritius, Aldabra, Comoros, and the Seychelles; certain in situ volcanic islands, such as Grande Comore, were formed no more than 0.5 ma [29] [30]. It is therefore reasonable to hypothesize that the island of Madagascar held large and stable populations of *Pteropus* that could have served as the source population for these smaller islands. Under this scenario, * P. aldabrensis*,* P. niger*, *P. s. seychellensis,* and * P. s. comorensis* would sensibly be nested within the *P. rufus * clade.

    While * P. s. comorensis* and *P. rufus* are distinct taxa on the basis of morphological characteristics [1], our population-level sampling suggests a more recent shared history than previously hypothesized. * Pteropus s. comorensis* and *P. rufus* may have evolutionary histories more similar to that found within smaller bodied western Indian Ocean bats. Several species of bats occur in both the Comoro archipelago and Madagascar, over 300 km away [14]. For two small bodied taxa, *Mops leucostigma* and *Chaerephon leucogaster*, there is only weak mitochondrial divergence between the regions [31] [32]. In contrast, two sister species of fruit bats in the genus *Rousettus* from Madagascar (*R. madagascariensis*) and the Comoros (*R. obliviosus*) are deeply divergent from one another at both mitochondrial and nuclear loci [33].

    Within each of the two species for which we have population level data, * P. s. comorensis* and *P. rufus, * we find only weak genetic differentiation. Among *P. s. comorensis * populations on the different islands within the Comoros, very little genetic diversity is explained by among island variation and F_ST_ is significantly greater than zero for only one population pair of intermediate geographic distance. No clear pattern of genetic differentiation has been found for several other species of bats from these islands including a smaller bodied fruit bat, *Rousettus oblivious* [33], and the molossid bat *Chaerephon pusillus* [34]. In contrast, a study of small bats of the genus *Miniopterus* from Anjouan and Grande Comore found much greater inter-island genetic divergence [35].

    We found moderate differentiation for the Malagasy species, *P. rufus*; shared haplotypes across the five sampled regions on Madagascar suggest ongoing gene flow and results from the AMOVA indicate that despite some differentiation, genetic diversity is largely explained by within population variation rather than among population differences. Nonetheless, pairwise F_ST_ values were significant for several population pairs (Table 3) and were notably high between Miandrivazo, in central Madagascar, and a more northern locality, Mahajanga, and also between Miandrivazo and the southeastern locality, Vangaindrano (Table 3). Miandrivazo may represent a geographically isolated region with corridors to the west and east connecting the other populations. While *P. rufus* do not appear to be panmictic across Madagascar, it is difficult to determine whether such patterns of differentiation are due to true barriers to gene flow or isolation by distance and genetic drift-gene flow equilibrium [36]. Previously, taxonomists segregated *P. rufus* into different subspecies, specifically the recognition of *P. r. princeps* named from the Tolagnaro region in southeastern Madagascar [37] and about 185 km south of our Vangaindrano sampling site. On the basis of the phylogeographic data presented herein, there is no evidence of divergence in the southeastern populations of * P. rufus*.

    Intraspecific patterns of genetic differentiation among other Malagasy bats vary considerably; some species are genetically panmictic throughout their distribution (e.g. *R. madagascariensis* [33]; * Triaenops rufus* [38]) whereas others show significant population genetic differentiation (e.g. *Myzopoda aurita* [39]; *T. furculus* [38]). Compared to *R. madagascariensis, * the other Malagasy pteropodid studied to date, *P. rufus* appears to have greater population structure. While we only include mitochondrial data for *P. rufus *and have limited geographic sampling in comparison to the *R. madagscariensis* study [34], our data suggest that patterns of population connectivity are not identical in these two fruit bats.

    The recent recolonization of *P. niger* on the Mascarene island of La Réunionindicates that there is considerable capacity of * Pteropus* bats in the western Indian Ocean to move among islands and across open expanses of water. Two species, *P. niger* and * P. subniger*, formerly inhabited this island but were extirpated by human hunting pressures [40]. The former species was able to maintain populations on Mauritius, and after an absence of at least 200 years on La Réunion, it has recolonized (Jean-Michel Probst, pers. comm.), traversing the ca. 170 km between these two islands. It is conceivable that long distance dispersal among islands of the Comoro archipelago and Madagascar occurs with some regularity, providing the opportunity for inter-island exchange of zoonotic diseases. However, such dispersal events may be rarer among other western Indian Ocean * Pteropus*.

## Conclusions

With the addition of multiple samples per species, it is clear that forms of western Indian Ocean *Pteropus*, as classically defined, have recently diverged from one another and that their species-level taxonomy may be in need of revision. Moreover, despite significant measures of intraspecific population genetic differentiation, *Pteropus* may be capable of long distance movements connecting populations across broad geographic distances. Including rapidly-evolving independent nuclear markers in future studies will undoubtedly provide insight into patterns of differentiation within and among species of *Pteropus* bats. Such data will guide sound taxonomic changes associated with the species limits of western Indian Ocean *Pteropus* and will also serve as an independent estimate of genetic connectivity that includes both maternal and paternal lineages.

## Acknowledgments

On Madagascar, we are grateful to the Direction des Eaux et Forêts and Association National pour la Gestion des Aires Protégées, and in the Comoros, to Yahaya Ibrahim of the Centre National de Documentation et de Recherche Scientifique and Ishaka Saïd of Action Comores for aid in numerous ways, including permission to collect specimens. We acknowledge Eddy Rakotonandrasana, Fanja Ratrimomanarivo, Manuel Ruedi, and Nicole Weyeneth for their aid with fieldwork, and Jean-Marc Reynes and Catherine Iehlé, formerly of the Institut Pasteur de Madagascar, for the collection of the *Pteropus *ear clips. We thank Brian Pope at the Lubee Bat Conservancy for his assistance, Teresa Ai, and Jonathan Schwartz for help with molecular data collection, the Duke Institute for Genome Science and Policy, and the Duke Shared Cluster Resource.

## Funding information

Conservation International (CABS), John D. and Catherine T. MacArthur Foundation, and the Volkswagen Foundation have generously supported field research associated with this paper. NSF award DEB-0516276 to SMG and ADY supported associated molecular research.

## Competing interests

The authors have declared that no competing interests exist.

****

## Supplementary materials

**Table S1.** Cytochrome-b sequences and associated GenBank numbers. SM refers to Supplementary Materials in O’Brien et al. [15].

**Table:** 

Species	GenBank Accession Number
*Acerodon celebensis*	GQ410231
*Cynopterus brachyotis*	AB046321
*Eonycteris spelaea*	AB062476
*Epomorphous wahlbergi*	DQ445706
*Ptenochirus jagori*	AB046325
*Pteralopex acrodonta*	FJ561376
*Rousettus amplexicaudatus*	AB046329
*Pteropus aldabrensis*	FJ561394
*Pteropus conspiculatus*	FJ561378
	FJ561379
	FJ561380
*Pteropus dasymallus*	AB042770
	NC002612
*Pteropus giganteus*	FJ561381
	SM [15]
*Pteropus hypomelanus*	FJ561382
	FJ561383
*Pteropus livingstonii*	FJ561384
	SM [15]
*Pteropus niger*	FJ561385
	SM [15]
	SM [15]
*Pteropus poliocephalus*	FJ561387
*Pteropus pumilus*	FJ561388
	FJ561389
	FJ561390
*Pteropus rodricensis*	FJ561391
	FJ561392
*Pteropus rufus*	AB085732
*Pteropus scapulatus*	AF321050
	NC002619
	FJ561377
*Pteropus seychellensis comorensis*	FJ561395
	FJ561396
	FJ561397
	FJ561398
*Pteropus seychellensis seychellensis*	FJ561399
	FJ561400
*Pteropus vampyrus*	AB062475
	AB046326
	FJ561401
	FJ561402
	FJ561403
*Pteropus voeltzkowi*	FJ561404
	FJ561405
